# Realization of Rectangular Artificial Spin Ice and Direct Observation of High Energy Topology

**DOI:** 10.1038/s41598-017-14421-w

**Published:** 2017-10-25

**Authors:** I. R. B. Ribeiro, F. S. Nascimento, S. O. Ferreira, W. A. Moura-Melo, C. A. R. Costa, J. Borme, P. P. Freitas, G. M. Wysin, C. I. L. de Araujo, A. R. Pereira

**Affiliations:** 1 Laboratory of Spintronics and Nanomagnetism (LabSpiN), Departamento de Física, Universidade Federal de Viçosa, 36570-000 Viçosa, Minas Gerais Brazil; 2 Departamento de Física, Universidade Federal de Ouro Preto, 35931-008 João Monlevade, Minas Gerais Brazil; 3 Brazilian Nanotechnology National Laboratory (LNNano), Brazilian Center for Research in Energy and Materials (CNPEM), Zip Code 13083-970 Campinas, Sao Paulo Brazil; 4 INL-International Iberian Nanotechnology Laboratory, 4715-330 Braga, Portugal; 5 Department of Physics, Kansas State University, Manhattan, KS 66506-2601 USA; 6 Instituto Federal do Espírito Santo, Alegre, 36570-900, Espírito, Santo 29520-000 Brazil

## Abstract

In this work, we have constructed and experimentally investigated frustrated arrays of dipoles forming two-dimensional artificial spin ices with different lattice parameters (rectangular arrays with horizontal and vertical lattice spacings denoted by *a* and *b* respectively). Arrays with three different aspect ratios *γ* = *a*/*b* = $\sqrt{{\bf{2}}}$, $\sqrt{{\bf{3}}}$ and $\sqrt{{\bf{4}}}$ are studied. Theoretical calculations of low-energy demagnetized configurations for these same parameters are also presented. Experimental data for demagnetized samples confirm most of the theoretical results. However, the highest energy topology (doubly-charged monopoles) does not emerge in our theoretical model, while they are seen in experiments for large enough *γ*. Our results also insinuate that the string tension connecting two magnetic monopoles in a pair vanishes in rectangular lattices with a critical ratio *γ* = *γ*_*c*_ = $\sqrt{{\bf{3}}}$, supporting previous theoretical predictions.

## Introduction

**Figure 1:**
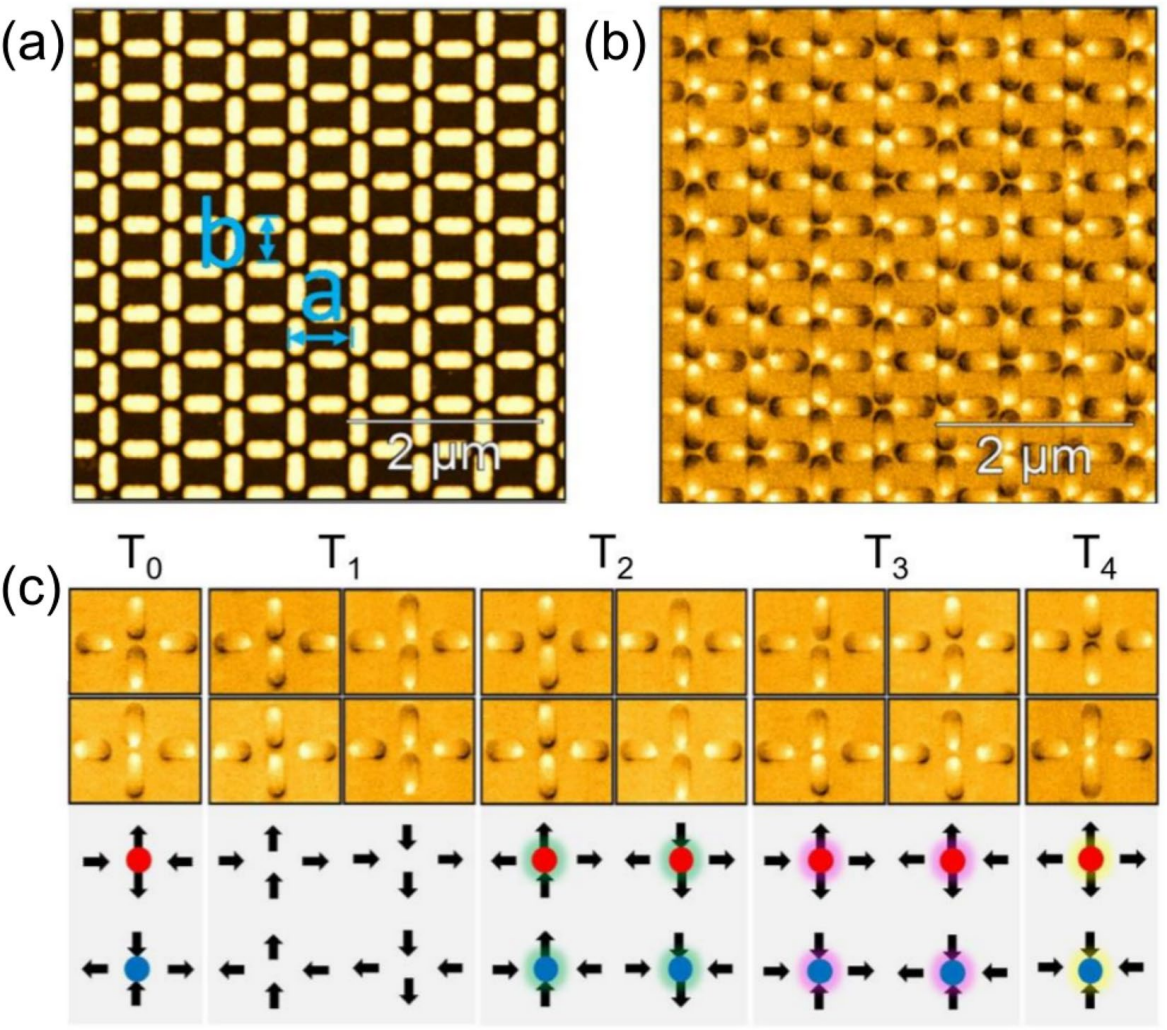
Artificial spin ice in a rectangular lattice. Consistent with other types of geometry (square, kagome etc), the ground state of a rectangular spin ice also obeys the ice rule in all vertices, which, in the present case, dictates that two spins must point-in and the other two must point-out. Excited states violate of the ice rule. The particular array shown here has the aspect ratio $\gamma =a/b=\sqrt{2}$. (**a**) Atomic force microscope topography of a typical sample for $\gamma =\sqrt{2}$. (**b**) Picture from the magnetic force microscope of single domain permalloy magnetic nanoislands (300 *nm* × 100 *nm* × 20 *nm*). Bright and dark ends of each elongated nanoisland indicate the opposite poles and give the direction of the magnetic moment of the islands. (**c**) The five possible topologies in this system. The circles in some vertices represent magnetic charges. We remind that the ground state *GSQ* is formed by the topology *T*_0_, while the ground state *GSM* is formed by topology *T*_1_.

Recently, the study of materials with frustrated interactions has received a lot of attention in an attempt to understand new states of matter^1–9^. The main problem concerning the experimental investigation of the properties of these structures is to find natural materials (in two and three dimensions), which not only clearly exhibit frustration but also provide reproducible results and adequate control for measurements. It is not such a simple task. An alternative path was provided by techniques of nanotechnology, in which artificial materials can be built with desirable properties and attributes in order to permit the materialization of a large variety of different sorts of geometrical frustration^10,11^. Especially, artificial spin ices in several different lattice geometries are important examples^5,8,12–14^. They are two-dimensional (2*d*) arrays of elongated magnetic nanoislands, each containing an effective magnetic moment or spin (see Fig. 1) that mimics natural three-dimensional (3*d*) spin ice materials^1–3^. However, such an artificial system in a 2*d* square lattice is not completely frustrated since the ice rule (in which two-spins must point-in and the other two must point-out in each vertex) is not degenerate (the two topologies that obey the ice rule have different energies^5,6^) and, therefore, the ice regime is not stabilized. Despite this, as in natural spin ices, artificial square ice (and even other kinds of artificial lattices) also supports quasiparticle excitations that are similar to magnetic monopoles^6,14–17^. Indeed, as shown by Castelnovo *et al*.^2^, excitations in natural spin ices behave like a magnetic monopole-antimonopole connected by a non-energetic but observable string (it is slightly different from the Dirac monopoles in which the string is also non-observable^18^). These objects and their strings were found by measurements from neutron-scattering experiments^19–21^. On the other hand, in general, monopole like excitations are of different types in artificial ice materials. For instance, the 2*d* artificial square ice supports excitations in which the oppositely charged monopoles occur connected by observable and energetic strings (a kind of Nambu monopole-antimonopole pair^16,22,23^). Therefore, it would be interesting to imagine and construct 2*d* artificial lattices whose monopole pair excitations would have a string tension that tends to vanish in such a way that, opposite magnetic charges would be effectively interacting only by means of the usual Coulomb law. However, in two dimensions, there is still additional entropic effects, which may cause some difficulties for this picture as we will remark later.

A recent theoretical proposal for vanishing the string tension was made to transform the square array into a rectangular one^17^. Inspired by this modified system, here we propose to realize an experimental study based on magnetic atomic force (*MFM*) measurements of the ground state and excited states of rectangular artificial spin ices (*RASI*). Denoting the horizontal and vertical lattice spacings of the rectangular array by *a* and *b* respectively (but always keeping the same dimensions for all magnetic bars), and defining a parameter (the aspect ratio) that controls the stretching of the lattice *γ* ≡ *a*/*b*, then, the theory^17^ predicts that the ground state suffers a transition at $\gamma =\sqrt{3}$ (or equivalently at $1/\sqrt{3}$ by interchanging *x* and *y* axes, or make *γ* ≥ 1 to avoid this ambiguity). Figure 1 shows an example of a fabricated rectangular array for $\gamma =\sqrt{2}$. In Fig. 1a,b, we present the sample topography and each island magnetic dipole (with topologies), respectively. In our investigation, we basically compare arrays with ratios $\gamma  < \sqrt{3}$ and $\gamma  > \sqrt{3}$ to the array having the critical value $\gamma ={\gamma }_{c}=\sqrt{3}$ (from now, dubbed *γ*_*c*_-array). For this comparison, we choose systems with lattice parameters having ratios equal to $\gamma =\sqrt{2}$ and $\gamma =\sqrt{4}=2$. Really, we clearly observe that such a deformation can tune the ratios of the interactions between neighboring elements resulting in different magnetic ordering of the system.

Before starting to discuss our work, it would be useful to describe earlier results about rectangular lattices. Indeed, theoretical calculations indicate that, for $1 < \gamma  < \sqrt{3}$, the ground state (denoted *GSQ*) has residual magnetic charges (but not magnetic moments) in all vertices, alternating from positive to negative in neighboring vertices. Such an idea of charge excess in the vertex centers is simplified (as discussed below) since this theoretical approach used the dumbbell model in the context of a system containing magnets that really have a length. Therefore, forgetting this trouble for a while, the total magnetic charge is zero. On the other hand, for $\gamma  > \sqrt{3}$, the ground state (denoted *GSM*) exhibits alternating residual magnetic moments (but not charges) in all vertices and, again, in this case, the total magnetic moment is zero. Exactly at the critical value $\gamma ={\gamma }_{c}=\sqrt{3}$, these two different configurations *GSQ* and *GSM* have the same energy and, therefore, the ground state at this particular *γ*_*c*_ becomes degenerate, suggesting a residual entropy at absolute zero temperature similar to what happens in natural^1,2^ and (3*d*) artificial^9,16,24^ spin ice materials. As a consequence, at ${\gamma }_{c}=\sqrt{3}$, the string tension connecating opposite magnetic charges tends to vanish and, in principle, the monopoles should become free to move. However, a description of free monopoles is not so simple in two dimensions. Really, in addition to the usual Coulomb interaction, there is an entropic effect that generates an attractive force between charges^22,24^, whose potential is proportional to the system temperature *T*. Its source comes from the fact that two monopoles should be attracted because there are more ways to arrange the surrounding dipoles in the lattice when they are close together (more space for disorder). This potential is given by *TlnR*, where *R* is the distance between a monopole and its antimonopole in a pair. Our experiments were accomplished at finite temperature and so, it is a huge challenge to find free monopole movement in these 2*d* arrays, even when the system parameters allow zero string tension. A possible way to avoid some protagonism of the entropic effect is to construct artificial spin ices in three dimensions. It consists of two sublattices of nanomagnets that are vertically separated by a small distance^9,16,24–26^ (a height offset *h*). The ice regime is found for a particular value of *h* = *h*_*c*_. Such a material was already produced and its authors have demonstrated unambiguous signatures of a Coulomb phase and that the local excitations are free magnetic monopoles evolving in an extensively degenerate, divergence-free vacuum^26^. Nevertheless, in 2*d*, things work in a very unusual way and, besides the usual attractive 3*d* Coulomb potential (1/*R*), the entropic attractive 2*d* Coulomb potential (Logarithmically with *R*) is an additional trouble for monopole movements. Further, even for the degenerate *γ*_*c*_-array, the two topologies *T*_0_ and *T*_1_ may coexist in the ground state and so, a purely Coulomb phase may be questioned; however, such a discussion is out of the scope of this paper.

**Figure 2:**
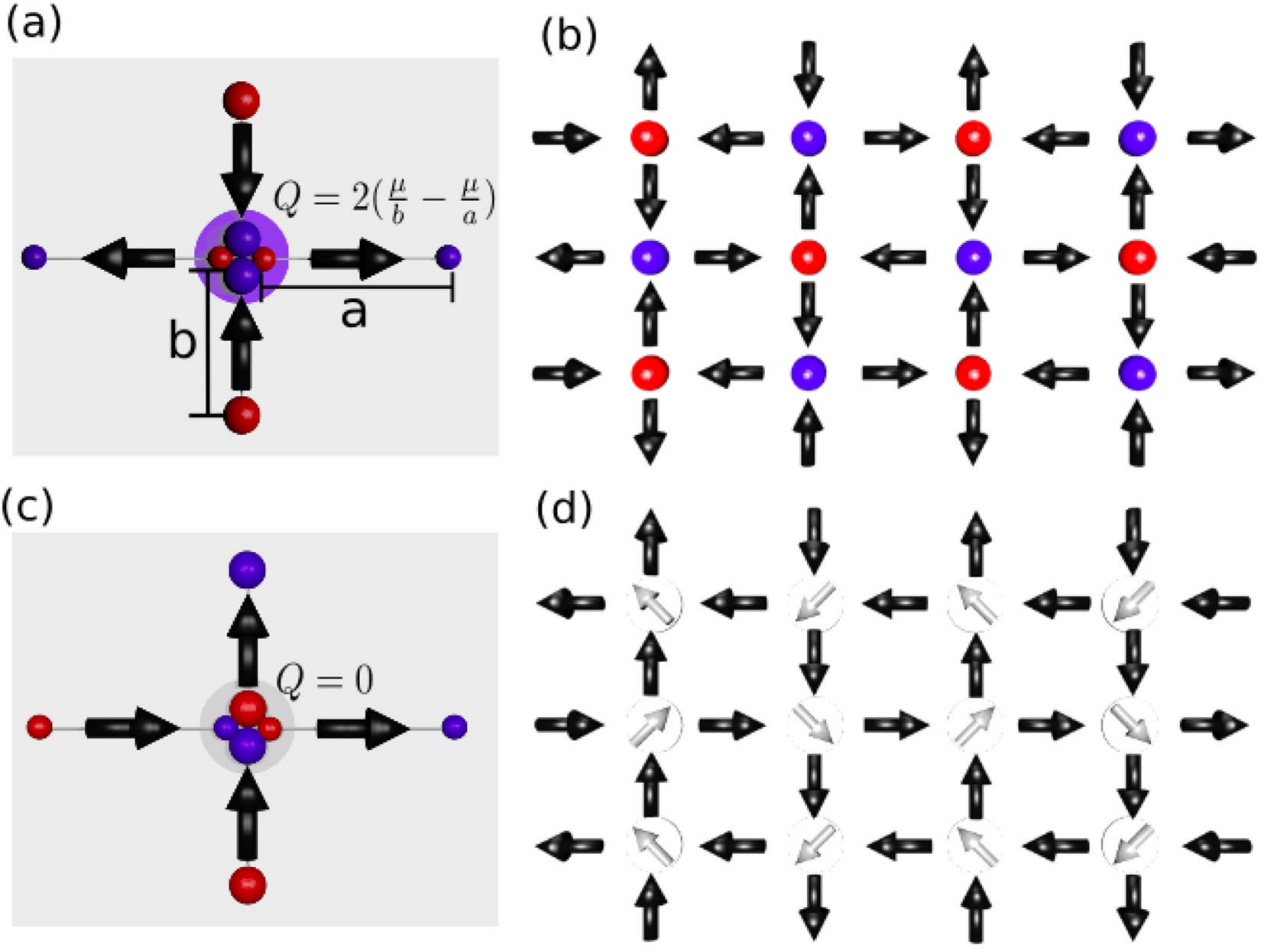
Detailed view of the ice rule in the topologies *T*_0_ and *T*_1_ in a rectangular ice, illustrating (with the use of the dumbbell model) how residual magnetic charges may occur even in the two-in, two-out ice rule. (**a**) In the *T*_0_ topology, the vertical dipole pairs as well as the horizontal dipole pairs are pointing both in or both out along opposite directions. Since the vertical magnetic bars are closer than the horizontal ones in each vertex, the resulting density of south (north) pole of the vertical dipoles are larger than the density of north (south) pole of the horizontal dipoles, inducing a residual charge, whose strength is estimated as |*Q*| = 2(*μ*/*b* − *μ*/*a*), where *μ* is the magnetic moment of the nanoislands (positive or negative values are associated with the opposite poles). Here, the magnetic charges were transported to the vertex center rather than localized at ends of the bars only to show schematically the differences between square and rectangular spin ices. However, in real *RASI*, this apparent excess of magnetic charge at the vertex centers presents a strong quadrupole moment. (**b**) Configuration of the ground state *GSQ* (with alternated residual charges in the vertices). (**c**) In the topology *T*_1_, the vertical dipole pairs as well as the horizontal dipole pairs are pointing along parallel directions. It means that the density of south (north) pole cancels with the density of north (south) pole vertically as well as horizontally, resulting in zero magnetic charge. Therefore, for the *T*_1_ topology, there is only a residual dipole (pointing out along the diagonal direction) in every vertex. (**d**) Configuration of the ground state *GSM* (with alternated residual magnetic moments in the vertices).

Differently from the planar square lattice (which has four distinct topologies^5^ for the four spins meeting at each vertex), the rectangular lattice exhibits five topologies^17^: *T*_0_, *T*_1_, *T*_2_, *T*_3_, *T*_4_ (see Fig. 1c). The first two (*T*_0_ and *T*_1_) obey the ice rule (two-in, two-out) with their energies depending on the parameter *γ*. For $1 < \gamma  < \sqrt{3}$, the energy of *T*_0_ is smaller than the energy of *T*_1_, while the contrary is valid for $\gamma  > \sqrt{3}$. It explains the ground states *GSQ* for $1 < \gamma  < \sqrt{3}$ and *GSM* for $\gamma  > \sqrt{3}$. Figure 2 illustrates how the topology *T*_0_, even obeying the ice rule (with two-in, two-out), has a residual magnetic charge (Fig. 2a), while the same does not occur for the topology *T*_1_ (Fig. 2c). Moreover, we also show the configuration of the ground state *GSQ* with its residual magnetic charges (alternating) at every vertex (Fig. 2b) and the configuration of the ground state *GSM*, which has residual magnetic moments (also alternating) at every vertex (Fig. 2d). However, we have to mention (to avoid some confusion) that the charge shown in Fig. 2 was constructed by the magnetic moment divided by the vertex-vertex lattice spacing, as used in natural spin ices^2^, where the atomic magnets can be treated as point dipoles. Of course, in artificial spin ices, the nanoislands are not point dipoles since they have a finite length. The magnetic charge is confined to the end of the nanoisland and, therefore, in these circumstances, the magnetic charge is defined by the moment divided by the length of the nanoisland^27^ as usually found in the literature. Although the dumbbell picture used by Castelnovo^2^ *et al*. for natural spin ices cannot be simply transposed to the artificial spin ice (since it does not describe the system quantitatively), it was used in Fig. 2 only to show (qualitatively) some differences between artificial square and rectangular spin ices. Therefore, in real *RASI*, in the ground state *GSQ*, the schematic excess of magnetic charge along the vertices should not be spherically symmetric. Indeed, as it can be easily seen in Fig. 2a, such a hypothetical central charge distribution has a strong quadrupole moment. In the most part of the subsequent text, we use the term topology even for the excitations identified with monopoles (i.e., *T*_2_, *T*_3_ and *T*_4_) instead magnetic charges.

## Results and Discussion

**Figure 3:**
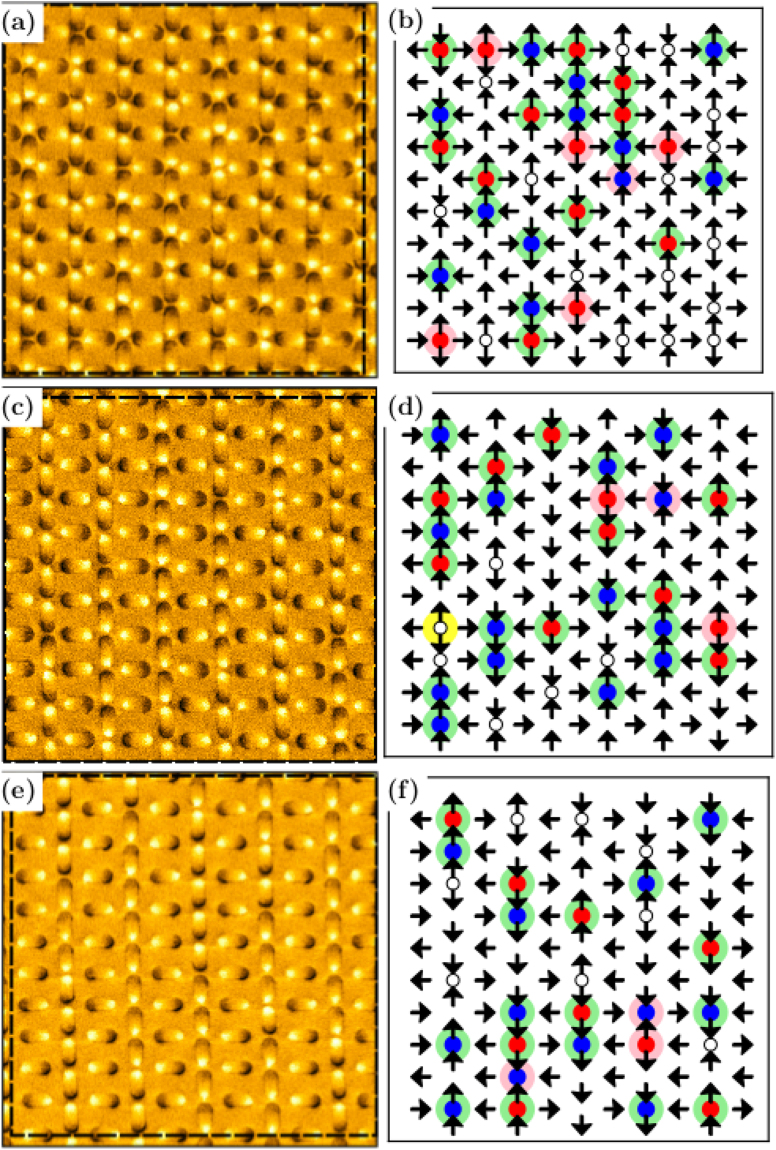
*MFM* results of artificial spin ice in a rectangular lattice and representations of magnetic charges observed in each vertex with: (**a**,**b**) $\gamma =\sqrt{2}$; (**c**,**d**) $\gamma =\sqrt{3}$; (**e**,**f**) $\gamma =\sqrt{4}$.

**Figure 4:**
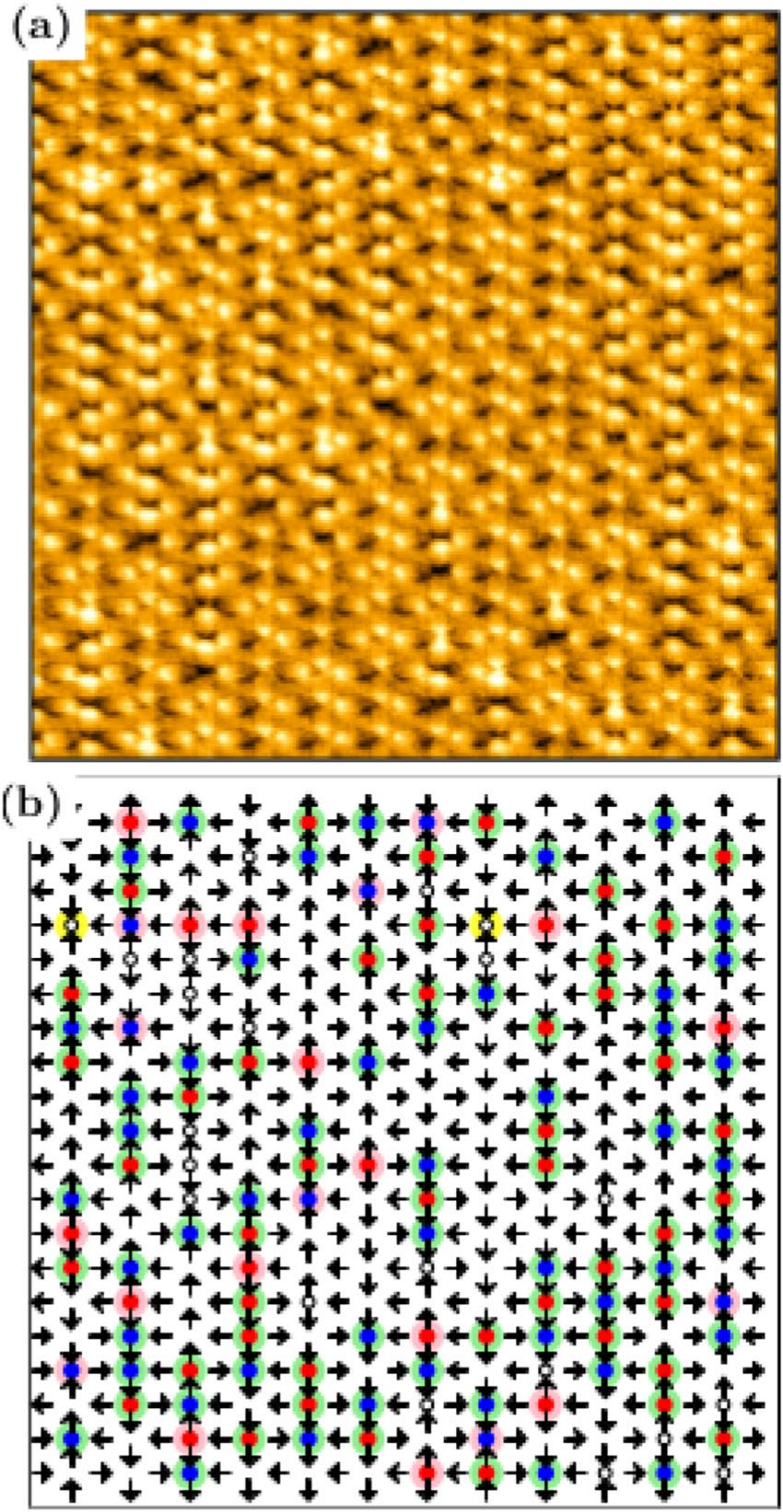
(**a**) Large area (100 *μm* ^2^) *MFM* view of a $\gamma ={\gamma }_{c}=\sqrt{3}$ sample and (**b**) magnetic moments and the topologies in each vertex, mapped computationally. Vertices without any circle are in the topology *T*_1_. Small white circles are residual charges (topology *T*_0_); white circles surrounded by a yellow circles are doubly-charged monopoles (*T*_4_ topology; here, one can see two of them, both in the four-in state). The other circles are single monopoles in topologies *T*_2_ and *T*_3_.

From the *MFM* measurements performed on the samples, we analyzed the distribution of topologies and total magnetization for three previously demagnetized *RASI* arrays studied ($a/b=\sqrt{2}$, $\sqrt{3}$ and $\sqrt{4}$), with a closer view presented in Fig. 3. To accomplish that, we computationally mapped the imaged dipole configurations and assigned a value *m*_*x*_ = ±1 or *m*_*y*_ = ±1 to each island moment, depending on the island magnetic orientation, as shown in Fig. 4. Table 1 summarizes the averaged experimental results, obtained after analysis. There is a very low total magnetization (in a range 0.03–0.10, close to zero), indicating a rather efficient demagnetization protocol. Additionally, the experimental data for the topology densities are very different from those expected for arrays with randomly oriented individual moments (*n*(*T*_0_)) = *n*(*T*_4_) = 12.5%) and (*n*(*T*_1_) = *n*(*T*_2_) = *n*(*T*_3_) = 25%); this is another indication that the demagnetization was successfully applied on the samples (for our purposes, the relevant parameters used in the experimental process to demagnetize the arrays were suggested by our Monte Carlo simulations as explained below; see also the Methods Section). Curiously, a few number of *T*_4_ topology (which has the highest energy) emerges for large enough *γ*, i.e., for $\sqrt{3}$ and $\sqrt{4}$ *RASI*. It is not seen for $\gamma =\sqrt{2}$. However, the direct experimental observation of this topology has never been predicted by our Monte Carlo calculations. In terms of real lattices and nanoislands, one possible reason to explain the appearance of *T*_4_ topology in experiments is the significant reduction of the energy scale between higher and lower energy of topologies (Fig. 1c).

**Table 1:** Summary of the experimental results for magnetization and topologies density for $a/b=\sqrt{2}$, $\sqrt{3}$ and $\sqrt{4}$.

*a*/*b*	T0	T1	T2	T3	T4
$\sqrt{2}$	0.16911	0.45456	0.25472	0.11731	0.00430
$\sqrt{3}$	0.09050	0.40643	0.38662	0.10651	0.00994
$\sqrt{4}$	0.12656	0.43257	0.35819	0.08020	0.00248

For the ground state topologies (*T*_0_ and *T*_1_), we found that the density of the *T*_1_ topology, as a function of *γ*, has a minimum at $\gamma ={\gamma }_{c}=\sqrt{3}$. The same can be said about the density of the *T*_0_ topology (but with values roughly four times smaller than the *T*_1_ topology). On the other hand, by taking into account the presence of monopole-antimonopole pairs in these systems (excitations above the ground state associated with *T*_2_ and *T*_3_ topologies), we notice that their density (the sum of *T*_2_ and *T*_3_ densities) is greater for rectangular lattices with the critical aspect ratio (*γ* = *γ*_*c*_) than that observed for others values of *γ*. These remarks can be seen in Table 1, which shows that the critical *γ*_*c*_-arrays exhibit the maximum number of monopoles possible. Note the explicit correlation between the smaller region of ground state and larger number of excitations when $\gamma =\sqrt{3}$ (i.e., the monopole density and the ground state topologies as a function of *γ* would present a maximum and a minimum, respectively, at *γ* = *γ*_*c*_).

The experimental observations were taken at room temperature (however, it is not important since these permalloy arrangements are expected to be athermal). This suggests that the different numbers of monopole pairs observed for different values of *γ* results from a purely geometrical effect, reinforcing the fact that monopoles could be more spontaneously generated in *γ*_*c*_-arrays. A simple argument could give some support to this picture: considering that the total energy of a pair depends also on the energy of the string connecting the monopole with its antimonopole^6^, then, a reasonable hypothesis for this geometrical influence on monopole number is that the string energy decreases as *γ* → *γ*_*c*_, corroborating previous theoretical results^17^, which predict very low string tension for *γ*_*c*_-arrays. Indeed, in Fig. 4, in a large section of a sample with *γ* = *γ*_*c*_, one can clearly observe a great quantity of monopole-antimonopole pairs (most of them with a monopole separated from its antimonopole by a distance equal to *a* or *b*) and also a small quantity of monopoles almost isolated.

**Figure 5:**
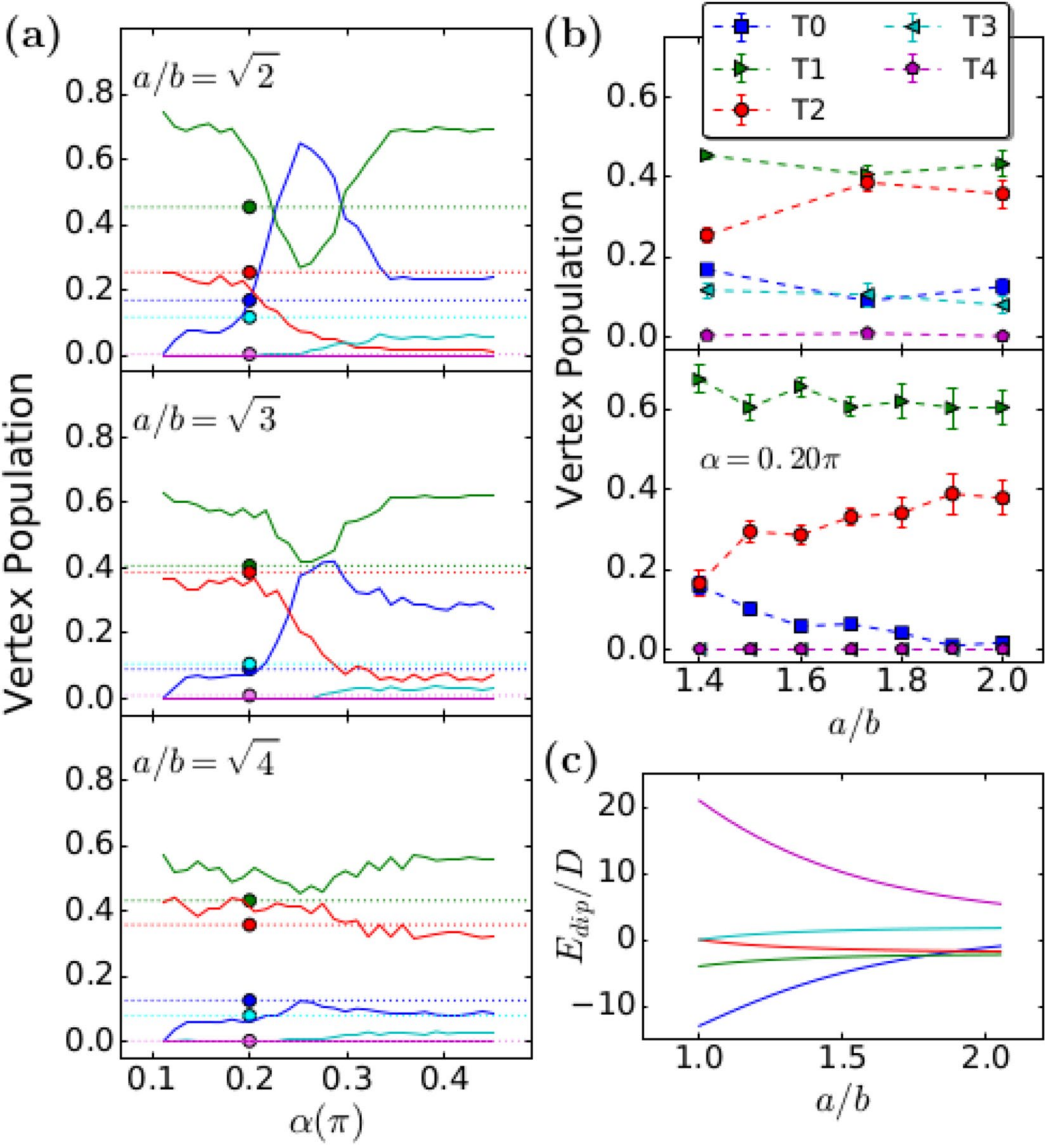
(**a**) Vertex population densities as functions of angle *α* between the demagnetizing field and the unitary cell along the *x*-axis for: (Top to bottom), $\gamma =\sqrt{2}$, $\gamma =\sqrt{3}$ and $\gamma =\sqrt{4}$. The *MC* simulations have a relatively good agreement with the experimental data for small *α* (0 ≤ *α* ≤ 0.2*π*). It is reasonable since the experiments were performed for *α* ≈ 0 (the exact value is not precise owing to the microstructure of the samples. We still notice that *α* = 0.2*π* is the best simulation result for comparison with the experimental data. Then, the colored circles, which represent the experimental points, are put around *α* = 0.2*π* only for guiding the eyes (dotted lines are also guide to eyes). (**b**) Vertex population densities as functions of *γ* for the demagnetizing field for experimental (top) and theoretical (bottom, with *α* = 0.2*π*) results. (**c**) Topology energies as functions of *γ*.

We also carried out Monte Carlo (*MC*) calculations using macro Ising spins for the island dipoles to compare with experiments. To be closer to the experimental situation described above, a demagnetization field is included in the simulations. This differs from the earlier calculations^17^, which do not consider external fields. Figure 5 shows the topology densities after having applied the demagnetization procedure in the *MC* simulations and its comparison with the topology densities measured by *MFM*. The theory indicates that the final topologies depend significantly on *α*, which is the angle that the external magnetic field is applied in relation to the larger lattice spacing (horizontal or *a*-axis in Fig. 3). It is expected mainly for *α* ≈ *π*/4, which affects spins in both vertical and horizontal directions. On the other hand, for *α* < 0.15*π* (*α* > 0.35*π*) the energetic flow occurs only on horizontal (vertical) dipoles. Of course, such behavior is a consequence of the fact that, if the external field is too oblique in relation to the horizontal dipoles, the projection of this field along the perpendicular dipoles will not be sufficient to overcome the islands’ switching barriers ${h}_{c}^{i}$, so the perpendicular dipoles will be frozen, i.e., they will maintain the initial configuration. We should remark that the *MC* simulations do not include the effects of thermal fluctuations, which might explain why the *T*_4_ topology is not reproduced by them. Perhaps even minor thermally induced fluctuations would be enough to help to produce the doubly-charged poles. Initially, samples are magnetized in a diagonal direction, which implies that the topology densities start with the values *n*(*T*_1_) = 1 and *n*(*T*_0_) = *n*(*T*_2_) = *n*(*T*_3_) = *n*(*T*_4_) = 0. In the range 0.15*π* < *α* < 0.35*π*, the energetic flow is more equally distributed through all the system. Really, Fig. 5a shows the vertex population as a function of *α* and one can easily see that, for all arrays investigated, the *T*_1_ topology density has a minimum while the *T*_0_ topology density has a maximum around *α* ≈ *π*/4, which is the center of the interval [0.15*π*, 0.35*π*]. Furthermore, just at *γ* = *γ*_*c*_, these two densities are nearly equal when *α* ≈ *π*/4. It is important to notice that, for *α* close to ~*π*/4, the demagnetization process tends to yield the systems to their ground states. For instance, when $\gamma =\sqrt{3}$, about 80% of the vertices obey the ice rule (equally distributed in topologies *T*_0_ and *T*_1_); for $\gamma =\sqrt{2}$, about 70% of the vertices are in topology *T*_0_, which is its ground state, while for $\gamma =\sqrt{4}$ there are about 60% of the vertices in its respective ground state topology (*T*_1_). In the last case, there is not an accentuated change of the number of *T*_1_ topology as a function of *α*. The results of Fig. 5a are very suggestive and they guided us to use a demagnetization angle *α* close to zero in our experiments as we really did. Actually, the *MC* simulations indicate that, in general, if one wants to observe a larger number of monopoles in the arrays, this experimental demagnetization protocol might be accomplished in small angles *α*. Following this suggestion, all experimental data presented in this work were obtained with *α* close to zero (see the section about Methods; in principle, this angle was chosen to be zero, but some misalignment must occur due to the relatively large size of the samples). Reminding that the experiments were performed by using a small *α*, then, we must compare the experimental data with the *MC* results only for relatively small values of this angle. In fact, Fig. 5a shows a reasonable agreement between theory and experiments for *α* in the range [0, 0.20*π*]. Moreover, the best agreement occurs when *α* = 0.2*π* in the simulations, which is the maximum value of the interval, but relatively small (In the figure, the colored circles represent the experimental data and they were placed at *α* = 0.2*π* only as a guide to the eyes). Even for the best resemblance theory-experiment, our simulations were not able to exhibit the *T*_4_ topology and yet, the density of the *T*_3_ topology is also very small, arising only for large enough *α*. The results for the other three topologies are almost identical to the experimental data (blue, green and red circles representing the vertex population for *T*_0_, *T*_1_ an *T*_2_ topologies, respectively). At this point, we can say that the highest topologies, which have higher energies, are responsible for the main contrast between our theory and experiments. Maybe the system sizes used in our calculations are too small to get good statistics for the topologies of low probabilities. Figure 5b shows the theoretical behavior of vertex population for a range of lattice spacings with fixed *α* = 0.20*π* (top) and the experimental behavior for the three lattices investigated (for small *α*; bottom). In overall, the theoretical results for the ground state topologies (*T*_0_ and *T*_1_) are in good qualitative and quantitative agreement with experimental data. However, theoretically, the *T*_0_ density goes slowly from approximately 0.20 for $\gamma =\sqrt{2}$ to almost zero (for $\sqrt{4}$), while experimentally (Table 1 and top of Fig. 5b), this density varies from 0.16 for $\gamma =\sqrt{2}$, decreasing to 0.09 for $\gamma =\sqrt{3}$ (similar to theoretical results) but, it turns to increase again to 0.12 for $\gamma =\sqrt{4}$. Therefore, there is an important qualitative difference between our simulations and experiments in the region $\gamma  > \sqrt{3}$. For the density of the *T*_1_ topology (green line), the *MC* simulations indicate that it becomes practically constant (around 0.60) as *γ* varies, while experimental data (see again the Table 1) remains almost constant with [*n*(*T*_1_)] varying near above 0.4. Furthermore, considering the monopole excitations (*T*_2_ and *T*_3_ topologies), we observe a good quantitative agreement between the *MC* simulations and experiments (Fig. 5b and Table 1) only for *T*_2_-type monopole (red line). For *T*_3_ topology (cyan line), the simulations lead to a very low density as compared to experiments. Despite the differences pointed out here, we can say that, in general, there is an overall qualitative (and even quantitative) agreement between the simple Ising spin model for magnetic nanoislands used in the simulations and our experimental data. These agreements become better in the region $1 < \gamma  < \sqrt{3}$. Finally, we have also calculated the energy of the topologies as a function of *γ* (see Fig. 5c). The calculations indicate that, independently of *γ*, the energy for creating *T*_3_ monopoles is bigger than the energy for creating *T*_2_ monopoles. It may explain the lower presence of *T*_3_ excitations around the lattice in both theoretical and experimental results. In addition, the energy of doubly-charged monopoles (*T*_4_ topology) is the biggest one (as expected), but it decreases relatively rapidly as *γ* increases. Such behavior, to some extent, justifies the direct observation of these *T*_4_ excitations in experiments for *γ* large enough ($\gamma =\sqrt{3}$ and $\sqrt{4}$, see Table 1).

In summary, we have experimentally and theoretically investigated two-dimensional artificial spin ices in rectangular lattices. Theoretical demagnetization scheme has suggested that the number of monopoles in the arrays is more significant only when the angle *α* between the demagnetization field and the direction parallel to *a* (here, the *x*-axis) is small. Then, our experimental data were obtained for *α* ≈ 0 (however, misalignments during the experimental demagnetization protocol is expected, effectively increasing this angle). The topology densities of the experimental samples (numerically counted from the *MFM* measurements realized in arrays with $a/b=\sqrt{2},\,\sqrt{3},\,\sqrt{4}$) were compared to the topology densities obtained theoretically by deadened sinusoidal external fields. The overall qualitative agreement between the simple theoretical model and experimental results is remarkable. A quantitative agreement is better achieved mainly when the demagnetizing field of the simulations is applied at an angle close to *α* = 0.20*π*. Therefore, in general, we can say that the experimental results corroborate the simple theory of Ising spin islands most used nowadays, but interestingly, topology *T*_4_ (doubly-charged monopole), which has the highest energy, could be seen only in experiments for lattices with large enough *γ*. Concerning this fact, *MC* simulations are able to give, at least, a route for this experimental visualization, showing that the energy of the *T*_4_ topology decreases considerably as *γ* increases (Fig. 5c). Of course, some disagreements between the theory developed here and experiments are to be expected in view of the exceedingly complex samples as compared to the simple theoretical approach. The behavior of the density of magnetic monopoles (topologies *T*_2_ and *T*_3_) is a purely geometrical effect, having a maximum at an intermediate array (*γ*_*c*_-array). Such a phenomenon may be associated with the fact that the string tension tends to vanish as *γ* → *γ*_*c*_, lending support to previous theoretical predictions^17^.

## Methods

For the fabrication of Permalloy nanoislands, a multilayer with composition *Si*/*Ta* 3 *nm*/*Ni*_80_ *Fe*_20_ 20 *nm*/*Ta* 3 *nm* was previously prepared by sputtering from tantalum (seed and cap layer) and alloyed permalloy target, on silicon oxide substrate. Then, the samples were covered with a 85 *nm* layer of *AR* – *N*7520.18 negative tone photoresist and pattered by electron lithography at 100 *kV* of acceleration voltage. After development, the samples were etched by ion milling at 20° from normal incidence, using secondary ion mass spectroscopy to detect the end of the process. An ashing in oxygen plasma was subsequently performed to remove the photoresist. The nanoislands dimensions of *l* = 300 *nm* and *w* = 100 *nm* leads to saturation magnetization 780 × 10^3^ *Am* ^−1^, giving a magnetic moment *μ* = 4.68 × 10^−16^ *Am* ^2^ per island. Then, for the *y*-axis lattice spacing *b* = 450 *nm* in our samples, the energy scale is *D* = *μ*_0_ *μ* ^2^/4*πb* ^3^ = 2.4 × 10^−19^ *J*. The *x*-axis lattice constant *a* ranged from 636–900 *nm* in such a way that we have investigated, by magnetic force microscopy (*MFM*), *RASI* arrangements with aspect ratios $a/b=\sqrt{2}$, $\sqrt{3}$ and $\sqrt{4}$. These systems were built on a area of 4 *mm* ^2^ and the *MFM* measurements performed in 25 and 100 *μm* ^2^ area, which enabled topologies density analysis in arrays of up 12 × 22 unit cell (528 islands). To find low energy configurations of the arrays of nanoisland dipoles, an experimental demagnetization protocol was carried out with a commercial demagnetizer. In this process, the magnetic field is switched from positive to negative values in the sample plane at a frequency of 60 *Hz*, as the samples are moved away from the coil center. We meant to move the samples in a direction parallel to *a* (*x*-axis); however due to the microstructure size of the samples, some misalignment can be expected. After the demagnetization process, the *MFM* measurements were carried out in four different regions of the samples, in order to improve the statistics. We have also done some Monte Carlo numerical calculations of low energy configurations to compare with the experimental data. To optimize this procedure, we have tested two different demagnetization protocols^12^. In the first, the sample is subjected to a sinusoidal magnetic field modulated by an exponential decay *h*(*t*) = *H*_*max*_ exp (−*t*) cos (2*π*60*t*), where *H*_*max*_ represents the field to saturate the sample. In the second, the magnetic field strength was stepped down (*H*_*max*_ − 0) in magnitude and switched in polarity with each step. However no substantial difference was found between the two protocols; so we adopted the first one to perform the experiments. In the simulations we have considered each magnetic nanoisland as a macro Ising spin. These spins are coupled via dipolar interactions. To obtain the evolution of the Ising spins under an external magnetic field, we have adopted the same procedure employed by Budrikis *et al*.^28^. In this consideration, one spin ${\overrightarrow{S}}^{i}$ can be flipped if the total field acting on it satisfies $({\overrightarrow{h}}_{ext}+{\overrightarrow{h}}_{dip}^{i})\cdot {\hat{S}}^{i} < -{h}_{c}^{i}$, where ${\hat{S}}^{i}$ represents a unit vector along the spin direction, ${\overrightarrow{h}}_{ext}$ is the external field, ${\overrightarrow{h}}_{dip}^{i}$ is the dipolar magnetic field produced by all spins of the lattice at the position where spin *i* is placed and ${h}_{c}^{i}$ is the island’s switching barrier. A perfect system is represented by a constant barrier while disorder can be implemented by taking ${h}_{c}^{i}$ from a Gaussian distribution with standard deviation *σ*. Here we consider disorder in the system to be absorbed into the dispersion of the switching barrier.
